# 2244: Objectively assessing student learning and effectiveness of an introductory educational program in clinical and translational research

**DOI:** 10.1017/cts.2017.167

**Published:** 2018-05-10

**Authors:** Elias M. Samuels, Thomas E. Perorazio, Brenda Eakin, Ellen Champagne, Marilyn Lantz

**Affiliations:** University of Michigan School of Medicine, Ann Arbor, MI, USA

## Abstract

OBJECTIVES/SPECIFIC AIMS: The first goal of this project is to test the reliability and validity of an objective structured clinical exam (OSCE) that was designed to assess competency in clinical and translational research. The second goal is to evaluate the impact of MICHR’s Summer Research Program on the participating trainee’s competency development. METHODS/STUDY POPULATION: The methodology used for this study was reviewed and exempted from oversight by the U-M Institutional Review Board (HUM00113293). The participants in the study include 17 pre-doctoral students in health professions programs at U-M who participated MICHR’s Summer Research Program. The Research OSCE was administered using a pretest, post-test design. The pretest was administered once during the 1st week of program in the Summer of 2016 and the post-test during the 10th week of the program. The Research OSCE was proctored and rated by trained staff members. We will assess the reliability of the Research OSCE using Generalizability Theory (Webb *et al*., 2006). And the construct validity of the Research OSCE will be tested using factor analysis and other statistical analyses. Growth in the competence of the trainees participating in the Summer Research program will be evaluated by testing for significant differences between their pretest and post-test scores. RESULTS/ANTICIPATED RESULTS: We anticipate that this study will show that the Research OSCE is a reliable competency assessment with proven construct validity. We also anticipate that the use of the Research OSCE will show the trainees participating in the Summer Research program experienced a gain in competence during the course of the 10-week program. DISCUSSION/SIGNIFICANCE OF IMPACT: This project uses a common and standardized testing approach. The primary goal of this project is to evaluate the reliability and validity of an OSCE to assess competency in clinical and translational research. It represents a new application for a well-studied testing method used extensively in the health professions to assess the clinical competency of health practitioners. This project will lead to a better understanding of (a) the reliability and validity of the Research OSCE designed to test research competency and (b) the effectiveness of the Summer Research Program curriculum in better preparing participants to conduct clinical and translational research. Showing how a specific competency assessment can be used for this purpose will provide the administrators, evaluators, and other stakeholders of clinical and translational research training programs with information that can be used to design more rigorous and relevant evaluations of their research training programs.

